# System Dynamics Model for Evaluating Socio-Economic Impacts of Different Water Diversion Quantity from Transboundary River Basins—A Case Study of Xinjiang

**DOI:** 10.3390/ijerph17239091

**Published:** 2020-12-05

**Authors:** Zhiying Shao, Fengping Wu, Fang Li, Yue Zhao, Xia Xu

**Affiliations:** 1 Business School, Hohai University, Nanjing 211100, China; shao_zy@hhu.edu.cn (Z.S.); lf@hhu.edu.cn (F.L.); zh_yyyyy@hhu.edu.cn (Y.Z.); 170212070004@hhu.edu.cn (X.X.); 2 National Engineering Research Center of Water Resources Efficient Utilization and Engineering Safety, Nanjing 210098, China

**Keywords:** Xinjiang, socio-economic impact, scenario analysis, system dynamics, transboundary river basins

## Abstract

With the rapid development of social economy and global climate warming, scarce transboundary water resources, as one of the basic resources for socio-economic development, have increasingly become the focus of basin countries. To investigate the socio-economic impacts of different water diversion quantity from transboundary river basins, we used a system dynamics (SD) model to reflect interactions between population, water resources, and socio-economic development, and applied it to a case study in Xinjiang to simulate its change tendency from 2011 to 2030 from the temporal dimension. Then, four water diversion quantity of transboundary river basins and four alternative socio-economic development patterns were designed to comprehensively evaluate these impacts of water diversion quantity change on the socio-economy of the region along the river under different socio-economic development patterns. The results indicate that (1) there was a positive correlation between water diversion quantity and the economic output value of the region along transboundary river basins, and the marginal benefit of transboundary water resources would decrease gradually; (2) considering the difficulty of water diversion from transboundary river basins and the protection of downstream water use and ecological health of transboundary river basins, we believe that increasing the transboundary water resources by 20% was more conducive to the sustainable development of Xinjiang’s socio-economy; (3) through the comparison of dynamic evolutions of socio-economic development and water impacts under four socio-economic development patterns, it is best for Xinjiang to plan its future development in the coordinated development of economic-resource scenario. Following this scenario, not only would the total output value of the socio-economy be better than other scenarios, but this also helps to alleviate the contradiction between the water supply and demand, which expected there would be a water shortage of 1.04 billion m^3^ in 2029 under 20% increase in water diversion quantity. Therefore, appropriate water diversion quantity, reasonable adjustment of industrial production growth rate, reduction of water consumption quotas of different industries and domestic water quota, and improvement of collection and treatment rate for sewage should be given priority in water resources management decision-making in Xinjiang or other arid regions along transboundary river basins.

## 1. Introduction

Water resources, as one of the natural resources that human society depends on, constitute an irreplaceable foundation of socio-economic development [1]. The demand for water resources increases with the rapid development of contemporary society, which leads to the increasingly prominent conflict between the supply and demand gap of global freshwater resources [2,3]. In particular, transboundary water resources, as an important part of freshwater resources, have increasingly become the focus of the basin countries to compete for. Currently, more than 150 transboundary rivers may trigger international water disputes [4]. The focus of disputes among basin countries is mainly on the water right allocation scheme because transboundary river basins involve two or even more upstream and downstream basin countries. It is, therefore, essential to develop and utilize transboundary river basins more reasonably and efficiently, which not only helps to alleviate the contradiction between supply and demand of local water resources, but also is conducive to maintaining stable relations between basin countries.

Transboundary water conflict in Central Asia is one of the most serious water resource issues in the world [5]. For example, the development and utilization of inland rivers has been difficult to meet the needs of local population growth, urbanization and industrial development in Xinjiang, China. In order to develop regional economy, the Chinese government has carried out appropriate development and utilization of the transboundary river basins (mainly Ili River and Irtysh River) through 635 Water Diversion Project, etc. Although the Chinese government has considered the use of water in the downstream areas and the ecological water demand of the whole basin, the media in Kazakhstan still put forward the Chinese water threat theory. Therefore, it is imperative to keep coordination of transboundary water resources and economy to solve water scarcity and water conflict issues in Xinjiang.

Previous studies on the relationship between water resources and socio-economic development were mainly measured by water resources carrying capacity (WRCC) [6,7]. This concept, extended from the concept of carrying capacity to the field of water resources, was used to appraise the mutual relationship between available water resources and socio-economic scale. Definitions of WRCC are far from unique because of different research purposes, research perspectives and research needs. Some scholars believed that WRCC refers to the maximum threshold of water resources that the environment can provide for sustainable socio-economic development [8] and human activities [9,10]; Other researchers considered WRCC is the maximum sustainable socio-economic and population scale that can be maintained on the basis of existing water resources and healthy water environment [11]. Jia et al. [12] once pointed out that the intensity and scale of socio-economic development should not exceed the carrying capacities of local water resources. Therefore, WRCC, as a criterion to measure sustainable development of water resources, was widely used to judge whether water environmental systems coordinate with socio-economic development in a specific region. The research scope on the relationship between water resources and socio-economic development has been rather abundant from the perspective of WRCC. For instance, Meng et al. [13] used a fuzzy comprehensive evaluation model to evaluate water resources carrying capacity in Tarim River Basin; Ding et al. [14] established a multi-objective model to analyze water ecological carrying capacity of urban lakes; Wang et al. [15] used system dynamics to appraise water resources carrying capacity of wetlands in Beijing; more studies were about regional water resources carrying capacity [16,17,18]. Besides, a few scholars introduced “coupling” to evaluate coupling coordination degree between water resources and socio-economic development. For example, Yu et al. [19] constructed a coordinated development degree model to analyze its coupling and coordinated development in Hubei Province.

To sum up, currently, from the perspective of research region, the above studies have focused nearly exclusively on inland rivers, urban lakes, and corresponding administrative regions. From the perspective of research method, we found that there is no unified evaluation method on the relationship between water resources and socio-economic development in academic. In some previous studies, a comprehensive evaluation indicator system was determined to quantitatively assess WRCC or coupling coordination degree between water resources and socio-economic development. However, these studies all adopted a static evaluation approach, and it is difficult to accurately and dynamically evaluate the relationships between water resources and socio-economic development due to complex and diverse influence relations in water environmental systems. In some other studies, the system dynamics model was established to comprehensively evaluate the relationships between the subsystems from the perspective of system, which can commendably overcome the above-mentioned deficiencies.

From the existing research, to our knowledge, there are still three important gaps: (1) the studies that have been performed on coordinate relationship between water resources and socio-economic sustainable development have focused nearly exclusively on inland rivers, urban lakes and corresponding administrative regions, and no specific analysis has been conducted on transboundary river basins and areas along them. (2) The above research on the relationship between water resources and economic development are all about evaluating WRCC, not considering inter-regional water diversion and the relationships between water diversion regions. (3) Little literature has paid attention to the socio-economic impacts of different water diversion quantity from transboundary river basins, and the dynamic changes of socio-economic development and water resources.

One of the primary missions of water resources management in Xinjiang is to keep balance between the water diversion quantity from transboundary river basins, socio-economic water use and socio-economic development, and this dynamic equilibrium relationship is a complex system problem. In this study, we focused on quantitatively assessing socio-economic impacts of different water diversion quantity from transboundary river basins based on system dynamics method to overcome the abovementioned deficiencies. By taking Xinjiang in China as a case study, we build a system dynamic simulation model with five subsystems, i.e., population, economy, water demand, water supply, and water shortage. Subsequently, four water diversion quantity from transboundary river basins and four socio-economic development patterns were designed to analyze the dynamic change trend of socio-economic development and water resources under different scenarios.

As discussed above, the main contributions of this research in the following points: (1) A systems dynamics (SD) model was employed to comprehensively simulate the interaction process of socio-economic development and water resources in Xinjiang, which is an arid area along transboundary river basins. (2) Socio-economic impacts of different water diversion quantity from transboundary river basins were evaluated. (3) Optimal water diversion quantity from transboundary river basins and socio-economic development pattern were determined by scenario analysis, which provide suggestions on water resources management in the context of basin countries for water resource managers and policy makers. This paper is organized as follows. Section 2 introduces the case study area and the acquisition of related data in the first place. Then, this section describes the method with main focus on the establishment and validation of the SD model in Xinjiang and different scenarios. Section 3 provides the simulation results under the different scenarios. Section 4 compares and discusses these simulation results and the optimal water diversion quantity from transboundary river basins. Section 5 presents the main conclusions and provides some suggestions for water resource managers and policy makers.

## 2. Methodology

This section firstly introduces the case study area and the acquisition of related data. Secondly, it describes the method and then establishes and verifies the SD model in Xinjiang. Finally, various scenarios are constructed and designed.

### 2.1. Study Area and Data Sources

#### 2.1.1. Site Description

The study area is Xinjiang (73° E–96° E, 34° N–48° N), which is a big province facing serious water scarcity issue [20]. The implementation of the great western development strategy has not only narrowed the development gap with other regions, but also intensified the pressure on local water resources, and the inland rivers have been unable to meet its socio-economic development needs. Accordingly, transboundary water resources play an important role in the socio-economic development of Xinjiang and alleviating the contradiction between supply and demand of local water resources.

The study area includes several transboundary river basins, and the development and utilization of transboundary river basins are mainly Ili River and Irtysh River [21]. Ili River is 1236 km long with a watershed area of 1.51 × 10^5^ km^2^, and it has about 22.8 billion m^3^ of water resources. This river in China is 442 km long with a watershed area of 5.6 × 10^4^ km^2^, the most abundant runoff river in Xinjiang. Irtysh River in China is 546 km long with a watershed area of 5.7 × 10^4^ km^2^. Its water volume is second only to the Ili River, ranking second in Xinjiang. According to the statistics of FAO, the surface water resources of Irtysh River in China are about 9.53 billion m^3^. The average annual surface runoff of these two transboundary river basins accounts for 1/3 of the total surface runoff in Xinjiang, while the water resource utilization rate is less than 1/4 of its surface runoff [22], which is far lower than the development and utilization rate of inland rivers in Xinjiang. Previous study has shown that China’s domestic utilization rate of transboundary river basins between China and Kazakhstan is less than 25% [23].

#### 2.1.2. Data Sources

Two main types of data were employed for this paper; namely, socio-economic data and water resources data, which were mainly collected from the Xinjiang Statistical Yearbook (2012–2019) and the Xinjiang Water Resources Bulletin (2011–2016). In addition, other information was collected from standard for domestic water consumption of urban residents GBT 50331-2016, Xinjiang’s discharge standard of rural domestic sewage treatment and China Urban Statistical Yearbook (2011–2018), spanning domestic water quota for residents (rural and urban), sewage discharge coefficient of residents and sewage treatment rate of sewage treatment plant. The socio-economic data included the population (rural and urban), urbanization rate and gross domestic products, while water resources data included the total volume of surface water resources, total volume of groundwater resources, recyclable water resources, industrial water use, daily domestic water for residents (rural and urban) and the volume of wastewater discharge.

### 2.2. System Dynamics Model

A systems dynamics (SD) model was employed to comprehensively evaluate the impact of transboundary water resources on the socio-economic development of Xinjiang in this study. SD, initially introduced by Forrester [24], was a well-established system simulation method for exploring complex dynamic feedback systems. Based on feedback theory, control theory and information theory, the SD approach predicts and simulates the relationship between subsystems through feedback behavior among variables from the perspective of system [25]. Comparing to the traditional methods, SD model can be applied to the dynamic, interacting and information feedbacks, and the feedback loop can be instantaneous or delayed [26].

Recently, SD model has been widely used in water resources management studies. Wei et al. [27] developed a simulation model to appraise the impact of different levels of environmental flow on socio-economy. Dawadi and Ahmad [28] evaluated the impact of water resources management under climate changes and population growth. Other applications of SD regarding water resources management include: sustainable development [29,30,31], reservoir operations [32,33], carrying capacity of water resources [16,34], water allocation [35,36], and water quality management [37].

### 2.3. Establishment of the SD Model

A system dynamic simulation model was applied to comprehensively simulate the interaction process of socio-economy and water resources in Xinjiang, which is to assess its socio-economic impacts of different water diversion quantity from transboundary river basins. This study chooses the period 2011–2030 as the research period, where historical data period is from 2011–2018, and the simulation time step is 1 year. A total of 5 subsystems are then used to analyze the socio-economic impacts of different water diversion quantity from transboundary river basins, namely population, economy, water demand, water supply, and water shortage, as described more in detail below. The main variables and equations used in this study are summarized in Table 1.

#### 2.3.1. Population Subsystem

**Figure 1:**
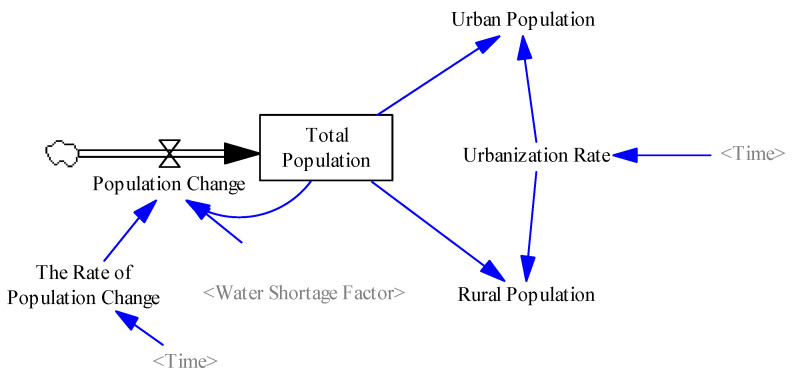
System dynamics flowchart of the population subsystem.

The population, which is one of the important components in socio-economic development, has a great impact on water resources [38]. In the population subsystem, the total population is determined by the population change, which is a rate variable leading to total population change (Figure 1). The population subsystem interacts with the other subsystem that profoundly affect the socio-economy and the water shortage. For instance, population growth increased the consumption of water resources, which will aggravate the local water supply and demand gap. On the contrary, water shortage limited population growth [39]. Therefore, the population change is affected by the population change rate and water scarcity factor. In addition, the total population is composed of the rural population and the urban population, which is determined by the auxiliary variable, i.e., the urbanization rate.

#### 2.3.2. Economic Subsystem

**Figure 2:**
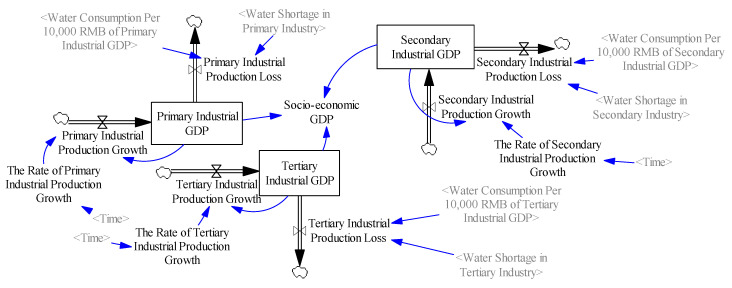
System dynamics flowchart of the economic subsystem.

The economic subsystem is an essential component of the overall system and aims to simulate the economic development trend under the influence of other subsystems (Figure 2). Xinjiang’s economic development lagged behind other regions due to its arid climate and remote location. According to the method of industrial classification, this subsystem is mainly composed of primary industry, secondary industry and tertiary industry, accounting for 25.9, 40, and 34.1% of Xinjiang’s real GDP, respectively. Agricultural water use accounts for more than 90% of the total socio-economic water consumption, which squeezes large amounts of water for ecological environment. In the economic subsystem, the output value of each industry is affected not only by its growth rate, but also by water resources. On the one hand, the stable development of economy depends on water resources, and its production activities need to consume a lot of water resources and discharge sewage into the natural environment. On the other hand, the deterioration of ecological environment and water shortage are both important factors restricting the scale of local socio-economic development [40]. In addition, the water resources utilization structure of various industries is closely related to economic development [41]. Therefore, the relationship between the economic subsystem and other subsystems is established, and the variables include the industrial GDP, the industrial production growth rate, water shortage, output value increase, and loss.

#### 2.3.3. Water Demand Subsystem

**Figure 3:**
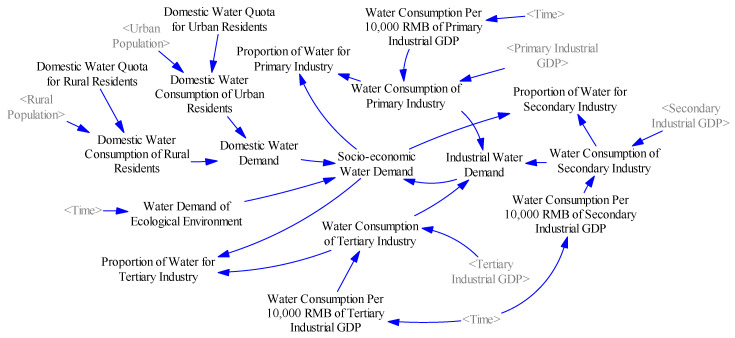
System dynamics flowchart of the water resource demand subsystem.

This subsystem simulates the demand of water resources for normal production and basic living requirements in Xinjiang, including industrial water demand, domestic water demand and water demand of ecological environment (Figure 3). Because industrial economic structure is one of the determinants of industrial water consumption [42], industrial water demand consists of water consumption of primary industry, secondary industry and tertiary industry, which reflect the quantity of water resources required by the production activities of various industries separately. Among them, the water consumption of secondary industry is the main source of wastewater. The domestic water consumption is the water consumed in the population’s daily life, which is also a source of wastewater. Considering that the water quota of urban residents is different from that of rural residents, the urban domestic water consumption and rural domestic water consumption are calculated separately. The water consumption of ecological environment mainly refers to the ecological water demand of urban green space and vegetation in Xinjiang according to the official data from Water Resources Bulletin.

#### 2.3.4. Water Supply Subsystem

**Figure 4:**
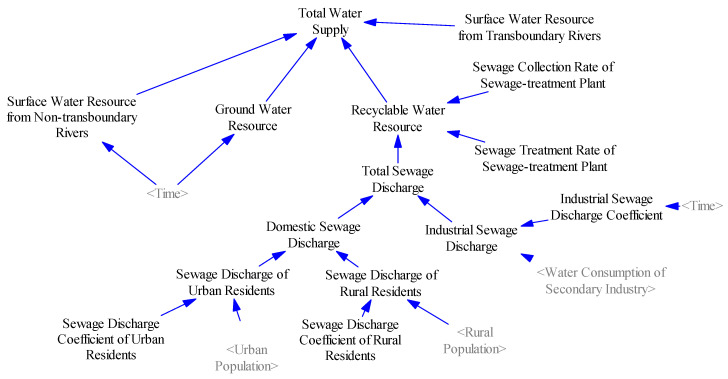
System dynamics flowchart of the water resource supply subsystem.

The purpose of the water supply subsystem is to reflect the supply capability of local water resources providing the fundamental material base for normal production and basic living requirements in Xinjiang. However, economic development, population growth and increased urbanization will aggravate the water supply burden [43,44], thereby intensifying the contradiction between the water supply and demand. In this study, the internal structure of this subsystem is divided into three parts: total surface water resources, total groundwater resources, and recyclable water resources (Figure 4). Considering that the inland rivers were not satisfied with local water demand, the transboundary river basins (i.e., Ili River and Irtysh River) have been properly developed and utilized. Therefore, in our study, the total surface water resources are composed of surface water resources in Xinjiang and water diversion quantity from transboundary river basins. A study by the Chinese Academy of Sciences shows that China’s utilization rate of the Ili River Basin is 36.2% [45], while the development and utilization of the Irtysh River Basin is mainly the completed “Yinejike” in 635 Water Diversion Project in Xinjiang, with an annual water diversion of 8.4 × 10^8^ m^3^. Recyclable water resources refer to the water resources that can be reused after the treatment of domestic sewage and industrial wastewater by the sewage treatment plant, which is determined by the collection rate and treatment rate for sewage of the sewage treatment plant.

#### 2.3.5. Water Shortage Subsystem

**Figure 5:**
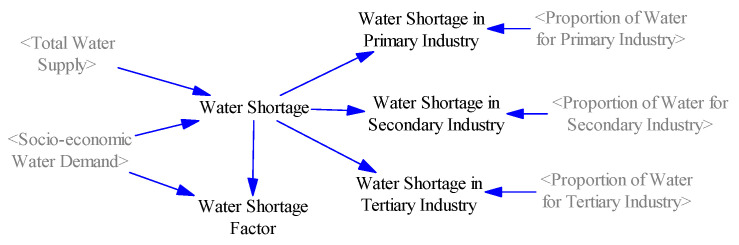
System dynamics flowchart of the water resource shortage subsystem.

The function of the water shortage subsystem is to reflect the relationship between supply and demand of water resources in the process of social-economic development, namely the gap between water supply and demand (Figure 5). This subsystem consists of two parts: industrial water shortage (including primary industry, secondary industry, and tertiary industry) and domestic water shortage. The industrial water shortage is obtained by multiplying the water demand ratio of each industry and the total water shortage. Water shortage factor, an auxiliary variable, is expressed by the ratio of water supply and demand gap to socio-economic water demand. To a certain extent, water shortage restricted the scale of economic development and population growth [46].

### 2.4. SD Model Validation

#### 2.4.1. Historical Data Test

Historical data test is to judge the consistency between the model system behavior and the actual behavior by comparing the gap between the simulation results and historical actual values, which is calculated by Equations (1) and (2):

(1) $$E=\left|\frac{{\hat{Y}}_{t}-Y_{t}}{Y_{t}}\right|$$

(2) $$AE=\frac{1}{n}\overset{n}{\underset{t=1}{\sum }}\left|\frac{{\hat{Y}}_{t}-Y_{t}}{Y_{t}}\right|$$

where E and AE are relative error and average relative error, *n* is the number of periods of historical data test, ${\hat{Y}}_{t}$ and $Y_{t}$ represent the simulation results and historical actual values at time *t*.

In this study, the level variables of total population, primary industrial GDP, secondary industrial GDP, and tertiary industrial GDP were selected to validate the model based on Equations (1) and (2) with the statistical data in Xinjiang in 2011–2018. The results of the relative error between the simulation results and historical actual values are shown in Table 2. It is generally considered that the absolute value of the relative error rate between the simulation results and historical actual values was less than 10%, which indicate that this model fitting effect is ideal [34]. In Table 2, the relative errors for all level variables were <6%, and the average relative error of all variables were <3.5%. These results indicate the SD model had good fitness in simulating the interactions and behaviors among subsystems in Xinjiang.

#### 2.4.2. Sensitivity Analysis

A sensitivity analysis is used to test the stability of the model system and analyze the sensitivity of the model system to the numerical adjustment by adjusting the parameters. The sensitivity of a variable relative can be calculated using Equation (3):

(3) $$S_{t}=\left|\frac{△Y_{t}}{Y_{t}}\times \frac{X_{t}}{△X_{t}}\right|$$

where $S_{t}$ is the sensitivity of the level variable *Y* to the parameter *X* at t time, $X_{t}$ and $Y_{t}$ are the value of the parameter *X* and the level variable *Y* at t time, $△X_{t}$ and $△Y_{t}$ are the variation of the parameter *X* and the level variable *Y* at t time. When the number of the level variable selected is not unique, the average sensitivity of parameter *X* can be calculated using Equation (4):

(4) $$S=\frac{1}{n}\overset{n}{\underset{t=1}{\sum }}S_{t}$$

where *n* and *S* represent the number of the level variable and the average sensitivity.

We selected four level variables, i.e., total population, primary industrial GDP, secondary industrial GDP, and tertiary industrial GDP and four constant variables, i.e., the rate of population change (PCR), the rate of primary industrial production growth (PIGR), the rate of secondary industrial production growth (SIGR), and the rate of tertiary industrial production growth (TIGR), which are more representative from the perspective of socio-economy. Based on the historical real data from 2011 to 2018, each selected constant variable parameter was manually ±10% in order to analyze its effect on the four level variables. The sensitivity analysis results were calculated by Equations (3) and (4), as shown in Table 3. Each constant variable parameter had different influence on different level variables. In addition, the average sensitivity of PCR and PIGR were <10% while the average sensitivity of SIGR and TIGR were 10–12%. Therefore, the sensitivity analysis results show that the system had low sensitivity to the constant parameters variables, and the model was robust.

### 2.5. Scenario Design

To promote the coordinated and sustainable development of water resources and socio-economy, different scenarios were proposed to analyze its dynamic relationship. In this study, each scenario involves two important aspects, i.e., water diversion quantity from transboundary river basins and socio-economic development pattern. Based on China’s contribution to these two transboundary river basins, we proposed different water diversion quantity scenarios by varying the present value of water diversion quantity from transboundary river basins by 0%, ±20% and ±40%. If the water diversion quantity from transboundary river basins is reduced by 20%, the water supply is 50.53 billion m^3^; If the water diversion quantity from transboundary river basins is reduced by 40%, the water supply is 48.71 billion m^3^, which is unable to meet the local normal production and basic living requirements of water resources, because the water demand is 50.60 billion m^3^ in Xinjiang, according to the analysis of water supply and demand balance in 2010 [47]. Thus, we abandoned the scenario of 40% reduction in water diversion quantity from transboundary river basins. The annual average water yield of Ili River and Irtysh River in China is 15.87 billion m^3^ [48] and 9.53 billion m^3^ [49], respectively. The water diversion quantity from transboundary river basins is 12.73 billion m^3^ when the water diversion quantity from transboundary river basins is increased by 40%, and China’s development and utilization of the basin accounted for about half of its contribution at this time. This shows that the scenario of 40% increase in water diversion quantity from transboundary river basins is in accordance with the norms of international law and international practice, according to China’s water demand and contribution rate to the basin [50]. Therefore, four scenarios of water diversion quantity from transboundary river basins were selected in this study, as shown in Table 4.

Additionally, four socio-economic development patterns were designed based on the development characteristics and development requirements of Xinjiang’s social economy; namely, business as usual (B1), economic development priority (B2), resource conservation priority (B3) and coordinated development of economic-resource (B4) and the detailed descriptions and quantitative assumptions are displayed in Table 5. B1 was the base scenario to estimate the future trend, which assumes that socio-economic development pattern will be maintained. B2 and B3 gave priority to economic development and resource conservation, respectively. B4 was a comprehensive and sustainable socio-economic development pattern, which took into account economic development and resource conservation, as well as its coordinate relationship, i.e., optimizing the industrial structure, moderately increasing the growth rate of the secondary and tertiary industries, and reducing domestic water quota and water consumption quotas of different industries. The four water diversion quantity from transboundary river basins and four socio-economic development patterns formed a total of 16 scenarios.

## 3. Results

The simulation results under the different scenarios are as follows.

### 3.1. Business as Usual-B1

**Figure 6:**
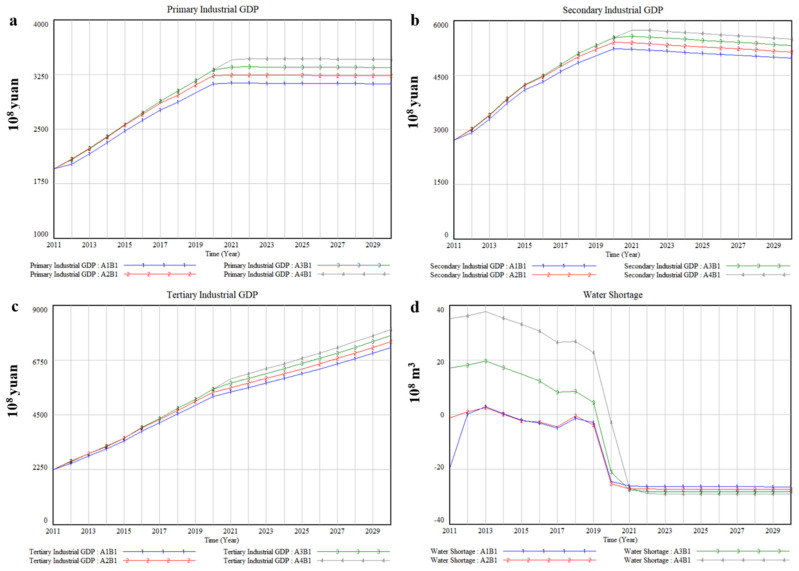
The simulation results of socio-economic and water impacts of 4 water diversion quantity from transboundary river basins under B1, (**a**) Primary Industrial GDP, (**b**) Secondary Industrial GDP, (**c**) Tertiary Industrial GDP, (**d**) Water Shortage.

The socio-economic development exhibited a slow upward annual trend. By 2030, the GDP of Xinjiang will reach CNY 1587.7 billion under A2B1. Among them, the primary industrial GDP and the secondary industrial GDP tend to be stable and slowly decline after reaching the peak, while the tertiary industrial GDP will have been increasing (Figure 6a–c). Table 6 lists average annual output value of various industries and its growth rate compared with A2 under B1, and the results illustrate that there is a positive correlation between the change of various industrial GDP and water diversion quantity from transboundary river basins. Taking the primary industry as an example, compared with A2, the average annual GDP will decrease by 3.46%, increase by 3.45%, and increase by 6.57%, respectively, when water diversion quantity from transboundary river basins is reduced by 20% (A1), increased by 20% (A3), and increased by 40% (A4). Moreover, the impact of different water diversion quantity from transboundary river basins on GDP of primary industry is higher than that of secondary industry and tertiary industry. The above results suggest that water resources are conducive to socio-economic development, which confirms that water resources contribute to economic development and poverty eradication from the perspective of simulation [51]. Furthermore, the growth of output value is positively correlated with water diversion quantity under A1-A2, while water diversion quantity will only affect the peak value of output value and the time to reach the peak value of output value under A3–A4. This could be because the increase in transboundary water resources leads to the decrease in water resources utilization efficiency.

In terms of water shortage, the increase in water diversion quantity from transboundary river basins cannot alleviate the contradiction between supply and demand of local water resources in essence. It can be seen from Figure 6d that the industry is almost always in a state of water shortage under A1–A2 and increasing water diversion quantity from transboundary river basins will only delay the occurrence of water shortage, which has not effectively improved local water scarcity issue. Specifically, water shortage is predicted to appear for the first time in 2020 under A3–A4, about 2.11 billion m^3^ and 283 million m^3^, respectively, and water shortage is estimated to reach 2.64 billion m^3^, 2.73 billion m^3^, 2.82 billion m^3^, and 2.91 billion m^3^, respectively, under different water diversion quantity in 2030. This shows that the problem of water shortage will be very serious if Xinjiang continues to develop with the current socio-economic development pattern.

### 3.2. Economic Development Priority-B2

**Figure 7:**
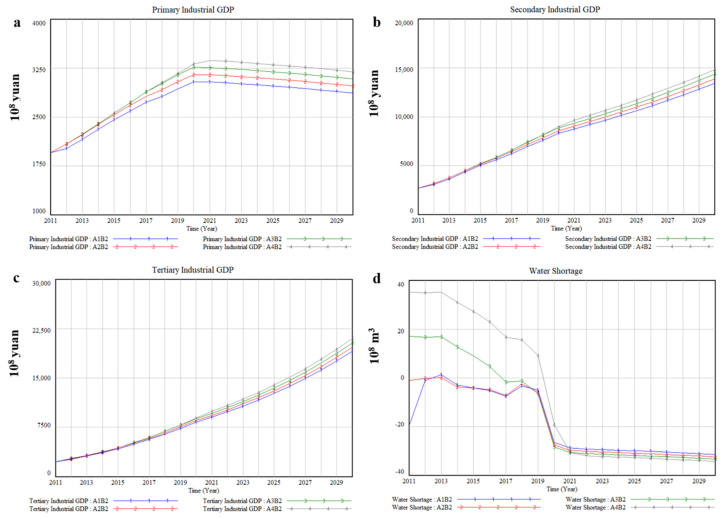
The simulation results of socio-economic and water impacts of 4 water diversion quantity from transboundary river basins under B2, (**a**) Primary Industrial GDP, (**b**) Secondary Industrial GDP, (**c**) Tertiary Industrial GDP, (**d**) Water Shortage.

In order to narrow the regional differences between it and other regions, Xinjiang has been pushing forward the great western development strategy. B2 aims to simulate socio-economic impacts of different water diversion quantity from transboundary river basins under the scenario of emphasizing economic development. The economic development of each industry is positively correlated with water diversion quantity from transboundary river basins, as shown in Figure 7a–c. Table 7 lists average annual output value of various industries and its growth rate compared with A2 under B2, and the results illustrate that there is a positive correlation between the change of various industrial GDP and water diversion quantity from transboundary river basins. Taking the primary industry as an example, compared with A2, the average annual GDP will decrease by 3.35% when water diversion quantity from transboundary river basins is reduced by 20% (A1), while the average annual GDP will increase by 2.78% and 4.86%, respectively, when water diversion quantity from transboundary river basins is increased by 20% (A3) and 40% (A4).

The socio-economic development will aggravate the contradiction between supply and demand of local water resources. As shown in Figure 7d, the water shortage exhibited an upward annual trend from 2019. The water shortage is predicted to reach 2.16 billion m^3^, 2.76 billion m^3^, 2.85 billion m^3^, and 1.92 billion m^3^ under different water diversion quantity from transboundary river basins in 2020. While the water shortage is predicted to reach 3.15 billion m^3^, 3.25 billion m^3^, 3.34 billion m^3^, and 3.44 billion m^3^ under different water diversion quantity from transboundary river basins in 2030.

### 3.3. Resource Conservation Priority-B3

**Figure 8:**
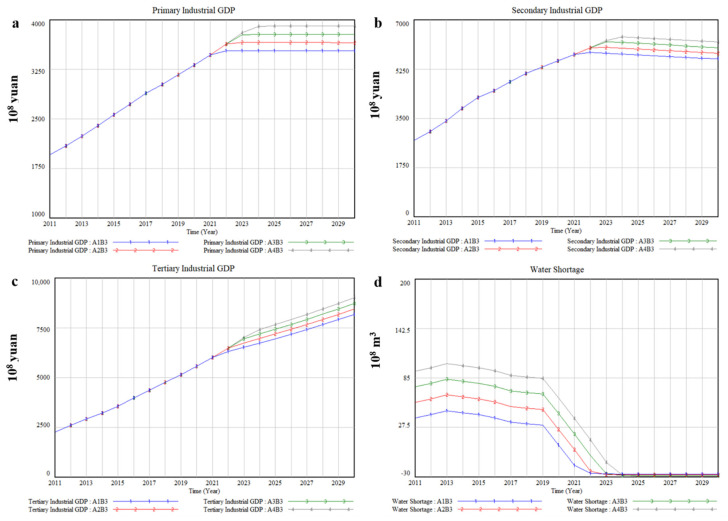
The simulation results of socio-economic and water impacts of 4 water diversion quantity from transboundary river basins under B3, (**a**) Primary Industrial GDP, (**b**) Secondary Industrial GDP, (**c**) Tertiary Industrial GDP, (**d**) Water Shortage.

Xinjiang is not only one of the most arid areas in China, but also one of the few extreme arid climate regions in the world. B3 refers to reducing the demand of water resources and increasing the supply of water resources from the perspective of secondary utilization, so as to alleviate the pressure of local water resources as much as possible. We try to change the parameters by reducing domestic water quota and water consumption quotas of different industries but properly improving the collection rate and treatment rate for sewage of the sewage treatment plant. The simulation results are shown in Figure 8a–d. In 2030, it is estimated that the total social and economic output value will be about CNY 1730.2 billion under A1. Table 8 lists average annual output value of various industries and its growth rate compared with A2 under B3, and the results illustrate the impact of water diversion quantity from transboundary river basins on tertiary industry is higher than that of primary industry and secondary industry. More concretely, compared with A2, the average annual GDP will decrease by 1.96% when water diversion quantity from transboundary river basins is reduced by 20% (A1), while the average annual GDP will increase by 1.78% and 3.42%, respectively, when water diversion quantity from transboundary river basins is increased by 20% (A3) and 40% (A4).

As shown in Figure 8d, the increase in water diversion quantity from transboundary river basins and resource conservation priority scenario slightly alleviate local water scarcity issue. Water shortage will occur under A2 in 2022, about 2.25 billion m^3^. Under A1 and A4, water shortage will occur one-year earlier or one-year later. While under A3, water shortage will occur in 2022, only about 529 million m^3^.

### 3.4. Coordinated Development of Economic-Resource-B4

**Figure 9:**
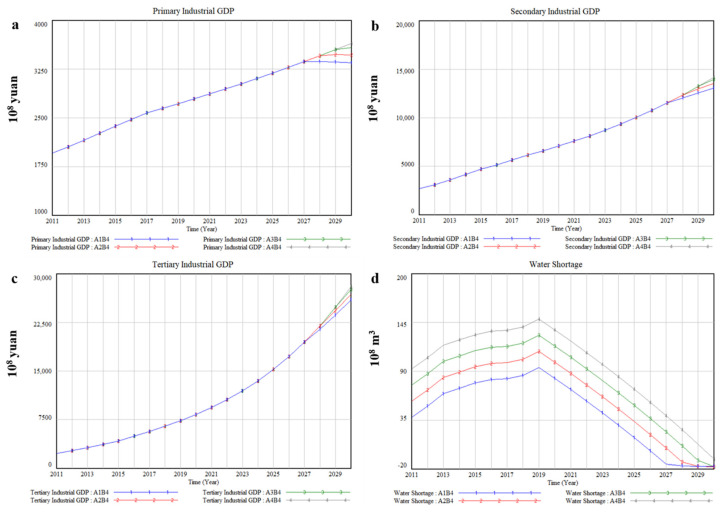
The simulation results of socio-economic and water impacts of 4 water diversion quantity from transboundary river basins under B4, (**a**) Primary Industrial GDP, (**b**) Secondary Industrial GDP, (**c**) Tertiary Industrial GDP, (**d**) Water Shortage.

The socio-economic development pattern of coordinated development of economic-resource (B4) was put forward in order to promote the coordinated development of regional economy in Xinjiang. The primary industrial GDP tends to decline under A1-A2 in the later stage of the simulation, while other industrial GDP will show a steady growth trend (Figure 9a–c). Additionally, the change of industrial GDP will be positively correlated with water diversion quantity from transboundary river basins. Specifically, the primary industry is expected to increase to CNY 336.63 billion in 2029 and then decrease to CNY 334.75 billion in 2030 under A1; the primary industry is expected to increase to CNY 347.48 billion in 2028 and then decrease to CNY 346.67 billion in 2030 under A2. However, the industrial GDP will exhibit an upward trend when increasing water diversion quantity from transboundary river basins, and the growth rate of output value is determined by the increase in water diversion quantity. Table 9 lists average annual output value of various industries and its growth rate compared with A2 under B4, comparing industrial economic impacts of different water diversion quantity from transboundary river basins in detail. The results further show that the utilization efficiency of 40% increase in water diversion quantity is slightly lower than that of 20% increase in water diversion quantity, especially in the tertiary industry. Specifically, the annual output value will increase by 0.59% under A3 while the annual output value will increase by 0.79% under A4. In other words, the first 20% increase in the water diversion quantity generated 0.59% of the output value, while the second 20% increase in the water diversion quantity only generated 0.2% of the output value.

Moreover, the water diversion quantity from transboundary river basins is positively correlated with the water shortage, and it is expected to well alleviate the pressure of local water supply and demand (Figure 9d). The water shortage will occur in 2027 even under A1, with a shortage of 1.49 billion m^3^. When water diversion quantity from transboundary river basins increases by 20% and 40%, there will be water shortage in 2029 and 2030, respectively, with 1.04 billion m^3^ and 908 million m^3^.

## 4. Discussion

In this section, these simulation results are firstly discussed and compared under the different scenarios. It then determines optimal water diversion quantity from transboundary river basins.

### 4.1. Comparison of the Scenarios

Compared with previous research [15,16], four water diversion quantity from transboundary river basins and four socio-economic development patterns were combined into 16 scenarios in this study. The results of scenario analysis are discussed as follows:

The results from the B1 scenario indicate that local socio-economy would grow slowly, and the water supply and demand situation was not optimistic. Due to the huge pressure on water resources and low coordination between water resources and socio-economic development [52], increasing water diversion quantity from transboundary river basins also cannot be essentially improved local water supply pressure. Given these conditions, the current socio-economic development pattern may fail to satisfy the requirements necessary for sustainable socio-economic in Xinjiang, which is consistent with the results of other studies [16,27,53].

In the B2 scenario, compared with B1, the relationship between the economic development of various industries and water diversion quantity from transboundary river basins has not changed, still having a positive correlation (Figure 7a–c). Additionally, the economic growth rate was about three times that of B1. However, the economic development impacts of various industries under different water diversion quantity from transboundary river basins have changed, mainly manifested in the primary industry and the secondary industry; specifically, the primary industrial GDP showed a slow downward trend after reaching the peak while the secondary industrial GDP showed a rapid growth trend.

Although giving priority to economic development was conducive to narrowing regional differences in Xinjiang, it has exacerbated local water scarcity issue. Because water consumption will increase as economic growth [54]. In the B2 scenario, the water shortage was increased by an average of 19.89% more than that in the B1 scenario under different water diversion quantity from transboundary river basins. In terms of improving local water scarcity issue, the results show that the B3 scenario was more effective than the scenarios mentioned above. In the B3 scenario, the general trend of economic development of each industry was similar to that in the B1 scenario, but the total economic output value has increased. Specifically, by 2030, the total economic output value was estimated to be CNY 1730.2 billion under a 20% reduction in water diversion quantity, higher than that in the B1 scenario under a 40% increase in water diversion quantity, about CNY 1695.85 billion. Moreover, compared with B1, the economic growth rate of various industries was not affected by water diversion quantity from transboundary river basins (Figure 8a–c). Therefore, ameliorating the repetitive utilization rate of water resources and reducing domestic water quota and water consumption quotas of different industries are good solutions for alleviating the pressure of local water supply and water demand [16], the same is true in Xinjiang.

**Figure 10:**
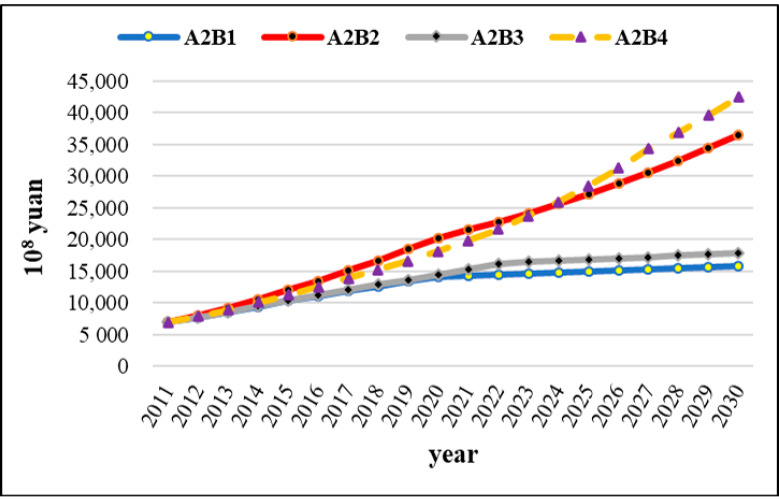
Evolution trend of total social and economic output value of the same water diversion quantity from transboundary river basins under 4 socio-economic development patterns.

In the B4 scenario, the total output value of social economy developed most rapidly. In consideration of the limited space, taking the A2 scenario as an example (Figure 10), the results show that the total socio-economic output value will have increased from CNY 691.89 billion in 2011 to CNY 4381.31 billion in 2030, which is better than the other three socio-economic development pattern scenarios. Therefore, in terms of the total socio-economic output value, the order of excellence of socio-economic development patterns under the same water diversion quantity from transboundary river basins is as follows: B4 > B2 > B3 > B1. Moreover, the B4 scenario better improved local water scarcity issue; specifically, even in the scenario of a 20% reduction in water diversion quantity, the water shortage is expected to occur for the first time in 2027, about 1.49 billion m^3^. However, by 2021, water shortage has appeared in different degrees under the other scenarios. In summary, the B4 scenario was optimal in all regards for the future socio-economic development in Xinjiang. This sufficiently indicates that optimizing the industrial structure, improving the utilization efficiency of water resources and strengthening the coordination of economy and resources can effectively alleviate the gap between water supply and water demand and realize regional sustainable development [53].

### 4.2. Optimal Water Diversion Quantity from Transboundary River Basins

Based on the comparative analysis of the above four scenarios of B1–B4, the B4 scenario was optimal for the future socio-economic development in Xinjiang. However, 40% increase in water diversion quantity from transboundary river basins reduced water resources utilization efficiency under the B4 scenario. In order to find the optimal water diversion quantity, we further narrowed the scope of the increase in water diversion quantity and subdivided it by every 5%. For every 5% increase in water diversion quantity from transboundary river basins, the annual average gross social and economic output value was shown in Table 10, which further confirmed that water diversion quantity was positively related to the economic development. Furthermore, every 5% increase in transboundary water resources has a decreasing trend, which shows that although increasing water diversion quantity from transboundary river basins was conducive to socio-economic development, the utilization efficiency of water resources of transboundary river basins decreased.

Currently, water resources allocation of transboundary river basins between China and Kazakhstan is still in the process of negotiation [55]. Considering the difficulty of water diversion from transboundary river basins, the protection of downstream water use and ecological health of transboundary river basins, the region along the transboundary river basins should not maximize the total benefit of transboundary water resources increase as the ultimate goal. The limit state of water diversion quantity of 635 Water Diversion Project is about 3 billion m^3^ in Xinjiang, and annual water diversion quantity of the completed “Yinejike” is about 840 million m^3^. The new water diversion project may affect the stable relationship between China and other basin countries and cause new transboundary water resources utilization conflicts. Therefore, the increase in water diversion quantity should be considered within the uncompleted “635” project—2.16 billion m^3^, which is between the 20–25% increase in water diversion quantity. Combination with Xinjiang’s Thirteenth Five-Year Plan for National Economic and Social Development, the economic growth index in the Plan has been exceeded when water diversion quantity was increased by 20%. At the same time, the shortage of water resources in this scenario has been improved, and the utilization efficiency of transboundary water resources was also considerable. Therefore, we believe that increasing the transboundary water resources by 20% was more conducive to the sustainable development of Xinjiang’s socio-economy.

## 5. Conclusions

This study established a composite SD model of social economy and water resources for evaluating socio-economic impacts of different water diversion quantity from transboundary river basins (A1, A2, A3 and A4). We validated this model and applied it to analyze the dynamic evolution of socio-economic impacts of different water diversion quantity from transboundary river basins and the relationship between them in Xinjiang in the period 2011–2030. Combination with four socio-economic development patterns (B1, B2, B3 and B4), this study then calculated, compared, and analyzed the dynamic evolution of the above 16 scenarios for finding the most suitable socio-economic development pattern and water diversion quantity from transboundary river basins. The key findings can be summarized as follows: (1) the B4 scenario will be optimal for the future socio-economic development of Xinjiang. Moderate economic growth rate, optimization of industrial structure, reduction of water consumption quotas of different industries and domestic water quota, and improvement of sewage collection and treatment rate are the main driving factors. (2) The increase in water diversion quantity from transboundary river basins will contribute to the local socio-economic development but the marginal benefit of transboundary water resources will decrease gradually. (3) Comprehensively considering various factors, increasing the transboundary water resources by 20% will be more conducive to realize its unit economic benefits.

The simulation and scenario design of this model aims to provide suggestions for water resource managers and policy makers in Xinjiang or other arid areas along transboundary river basins:

(1) It is necessary to optimize the industrial structure, reduce the water consumption quota and improve the utilization rate of water resources for arid regions. For example, agricultural products with large water consumption can be moderately reduced production, and the gap of local agricultural products can be solved through import.
(2) Chinese government should rationally develop and utilize transboundary water resources within the scope of international regulations and pay attention to the economic benefits of unit water resources. At the same time, they should pay attention to the compensation for the basin ecology and downstream after the development and utilization of transboundary river basins and establish a good cooperation relationship with the downstream.
(3) The relevant departments ought to do a good job in water storage projects and save the water resources in surplus years to alleviate the pressure of water supply and demand in the later period.

Water resource management in the context of transboundary river basin countries is a complex work, and this study has some limitations. Some relationships among the subsystems are simplified or not fully considered due to limited data. In the future, the relationships among the subsystems will be deeply analyzed to build a more comprehensive SD model for water resources management in the context of transboundary river basin countries.

## Figures and Tables

**Table 1:** The main variables and equations of the model.

No.	Variables	Equations	Units
1	Total population	=INTEG (Population Change, 22.0871)	10^6^ people
2	Population change	=Total population × Rate of Population Change × (1 − Water Shortage Factor)	10^6^ people
3	The rate of population change	=WITHLOOKUP {Time, [(2011, 0) − (2030, 10)], (2011, 0.012), (2012, 0.011), (2013, 0.014), (2014, 0.015), (2015, 0.027), (2016, 0.016), (2017, 0.019), (2018, 0.017)}	
4	Urban population	=Total population × Urbanization Rate	10^6^ people
5	Rural population	=Total population × (1 − Urbanization Rate)	10^6^ people
6	Urbanization rate	=WITHLOOKUP {Time, [(2011, 0) − (2030, 10)], (2011, 0.4354), (2012, 0.4398), (2013, 0.4447), (2014, 0.4607), (2015, 0.4723), (2016, 0.4835), (2017, 0.4938), (2018, 0.5091)}	
7	Socio-economic GDP	=Primary Industrial GDP + Secondary Industrial GDP + Tertiary Industrial GDP	10^8^ yuan
8	Primary industrial GDP	=INTEG (Primary Industrial Production Growth − Primary Industrial Production Loss, 1955.39)	10^8^ yuan
9	Primary industrial production growth	=Primary Industrial GDP × The Rate of Primary Industrial Production Growth	10^8^ yuan
10	The rate of primary industrial production growth	=WITHLOOKUP {Time, [(2011, 0) − (2030, 10)], (2011, 0.069), (2012, 0.07), (2013, 0.072), (2014, 0.068), (2015, 0.063), (2016, 0.06), (2017, 0.048), (2018, 0.047)}	
11	Primary industrial production loss	=IF THEN ELSE (Water Shortage in Primary Industry <0, ABS (10^4^ × Water Shortage in Primary Industry/Water Consumption Per 10,000 RMB of Primary Industrial GDP), 0)	10^8^ yuan
12	Water consumption per 10,000 RMB of primary industrial GDP	=WITHLOOKUP {Time, [(2011, 0) − (2030, 3000)], (2011, 2515.66), (2012, 2508.33), (2013, 2486.55), (2014, 2300.17), (2015, 2145.94), (2016, 1981.65), (2017, 1818.38), (2018, 1660.2)}	m^3^/10^4^ yuan
13	Socio-economic water demand	=Industrial Water Demand + Domestic Water Demand + Water Demand of Ecological Environment	10^8^ m^3^
14	Total water supply	=Surface Water Resource from Non-transboundary Rivers + Ground Water Resource + Recyclable Water Resource + Surface Water Resource from Transboundary Rivers	10^8^ m^3^
15	Recyclable water resource	=Total Sewage Discharge × Sewage Collection Rate of Sewage-treatment Plant × Sewage Treatment Rate of Sewage-treatment Plant	10^8^ m^3^
16	Water shortage	=Total Water Supply − Socio-economic Water Demand	10^8^ m^3^
17	Water shortage factor	=ABS (Water Shortage/Socio − economic Water Demand)	

**Table 2:** Historical data test results of the SD model (2011–2018).

		2011	2012	2013	2014	2015	2016	2017	2018
Total population (10^6^ persons)	Actual value	22.09	22.33	22.64	22.98	23.60	23.98	24.45	24.87
	Simulated value	22.09	22.35	22.60	22.91	23.26	23.88	24.26	24.72
	Relative error (%)	0.00	0.11	0.20	0.33	1.45	0.42	0.76	0.60
	Mean (%)	0.48							
Primary industrial GDP (10^10^ yuan)	Actual value	19.55	20.92	22.43	23.95	25.46	26.99	28.29	29.62
	Simulated value	19.55	20.86	22.32	23.93	25.55	27.07	28.56	29.70
	Relative error (%)	0.00	0.03	0.49	0.12	0.35	0.28	0.96	0.28
	Mean (%)	0.35							
Secondary Industrial GDP (10^10^ yuan)	Actual value	27.13	30.58	34.58	38.04	40.24	43.02	45.77	47.70
	Simulated value	27.13	30.16	33.99	38.44	42.29	44.58	47.44	50.09
	Relative error (%)	0.00	1.35	1.70	1.07	5.08	3.63	3.64	5.03
	Mean (%)	2.69							
Tertiary industrial GDP (10^10^ yuan)	Actual value	22.51	25.27	27.85	30.75	34.50	37.74	41.33	44.63
	Simulated value	22.51	25.88	29.06	32.02	35.35	39.53	43.05	46.80
	Relative error (%)	0.00	2.38	4.33	4.14	2.47	4.74	4.18	4.85
	Mean (%)	3.39							

**Table 3:** Results of sensitivity analysis of the SD model (2011–2018).

Level Variable	Constant Variable			
	PCR	PIGR	SIGR	TIGR
Total population	0.0510	0.0010	0.0005	0.0004
Primary industrial GDP	0.0039	0.1279	0.0900	0.0887
Secondary industrial GDP	0.0001	0.0045	0.3321	0.0043
Tertiary industrial GDP	0.0000	0.0008	0.0008	0.3756
Mean	0.0138	0.0336	0.1059	0.1173

**Table 4:** The changes of water diversion quantity from transboundary river basins.

Code	Description
A1	China reduces water diversion quantity from transboundary river basins by 20%
A2	China’s water diversion quantity from transboundary river basins remains unchanged
A3	China increases water diversion quantity from transboundary river basins by 20%
A4	China increases water diversion quantity from transboundary river basins by 40%

**Table 5:** The details of the different economic development scenarios.

Scenarios	Main Variables Needing Adjustment	Scenario Details
Business as Usual	–	The socio-economic development pattern maintains the status quo and all parameters are not adjusted.
Economic Development Priority	The Rate of Secondary industrial production growth The Rate of Tertiary Industrial Production Growth The Rate of Population Change	In view of the relatively backward economic development in Xinjiang, priority should be given to the development of regional economy. Emphasizes the development of secondary industry and tertiary industry to promote rapid economic growth. Speeds the rate of secondary and tertiary industrial production growth by 6% per year based on the Business as Usual pattern. Additionally, economic growth requires an increase in the working population, thus increasing the rate of population change by 6% per year.
Resource Conservation Priority	Collection Rate for Sewage Treatment Rate for Sewage Domestic Water Quota for Urban Residents Domestic Water Quota for Rural Residents Water Consumption Per 10,000 RMB of Primary Industrial GDP Water Consumption Per 10,000 RMB of Secondary Industrial GDP Water Consumption Per 10,000 RMB of Tertiary Industrial GDP	The focus of socio-economic development is to save resources and improve the utilization rate of water resources. Improve collection rate and treatment rate for sewage to 60% and 98%, respectively; Decrease both domestic water quota for urban residents and rural residents by 10%. Decrease water consumption per 10,000 RMB of different industries GDP by 10%.
Coordinated Development of Economic-resource	The parameters involved in the above scenarios	At the same time, we should give consideration to economic development and resource conservation and promote the coordinated development of regional economy with the help of industrial structure optimization. Appropriately reduce the rate of the primary industrial production growth and slowly increase the rate of the secondary and tertiary industrial production growth, especially the tertiary industry. Reduce the rate of the primary industrial production growth by 2%; Increase the rate of the secondary and tertiary industrial production growth by 3% and 5%, respectively. Other indicators refer to the changes of the Resource Conservation Priority pattern.

**Table 6:** Average annual output value of various industries and its growth rate compared with A2 under B1.

Industry Category	Scenario Category	Average Annual GDP (10^8^ Yuan)	Its Growth Rate Compared with A2
Primary industry	A1B1	2827.66	−3.46%
	A2B1	2924.01	0.00%
	A3B1	2987.86	3.45%
	A4B1	3038.40	6.57%
Secondary industry	A1B1	4582.85	−3.18%
	A2B1	4733.55	0.00%
	A3B1	4845.31	2.36%
	A4B1	4935.99	4.28%
Tertiary industry	A1B1	4994.56	−3.11%
	A2B1	5154.93	0.00%
	A3B1	5282.65	2.48%
	A4B1	5390.92	4.58%

**Table 7:** Average annual output value of various industries and its growth rate compared with A2 under B2.

Industry Category	Scenario Category	Average Annual GDP (10^8^ Yuan)	Its Growth Rate Compared with A2
Primary industry	A1B2	2729.73	−3.35%
	A2B2	2824.40	0.00%
	A3B2	2902.78	2.78%
	A4B2	2961.80	4.86%
Secondary industry	A1B2	8160.71	−3.14%
	A2B2	8425.23	0.00%
	A3B2	8668.40	2.89%
	A4B2	8871.17	5.29%
Tertiary industry	A1B2	9189.65	−3.07%
	A2B2	9480.41	0.00%
	A3B2	9756.88	2.92%
	A4B2	9996.16	5.44%

**Table 8:** Average annual output value of various industries and its growth rate compared with A2 under B3.

Industry Category	Scenario Category	Average Annual GDP (10^8^ Yuan)	Its Growth Rate Compared with A2
Primary industry	A1B3	3080.27	−1.76%
	A2B3	3135.50	0.00%
	A3B3	3185.00	1.58%
	A4B3	3230.06	3.02%
Secondary industry	A1B3	4987.94	−1.77%
	A2B3	5077.90	0.00%
	A3B3	5158.36	1.58%
	A4B3	5231.49	3.02%
Tertiary industry	A1B3	5453.76	−1.96%
	A2B3	5563.06	0.00%
	A3B3	5662.31	1.78%
	A4B3	5753.54	3.42%

**Table 9:** Average annual output value of various industries and its growth rate compared with A2 under B4.

Industry Category	Scenario Category	Average Annual GDP (10^8^ Yuan)	Its Growth Rate Compared with A2
Primary industry	A1B4	2793.124	−0.58%
	A2B4	2809.434	0.00%
	A3B4	2818.954	0.34%
	A4B4	2822.095	0.45%
Secondary industry	A1B4	7633.907	−0.76%
	A2B4	7692.522	0.00%
	A3B4	7727.472	0.45%
	A4B4	7739.187	0.61%
Tertiary industry	A1B4	10,847.8	−0.97%
	A2B4	10,953.01	0.00%
	A3B4	11,017.34	0.59%
	A4B4	11,039.32	0.79%

**Table 10:** Average annual gross output value of social economy and its growth rate under every 5% increase in water diversion quantity from transboundary river basins.

Growth Categories of Water Diversion Quantity from Transboundary River Basins	Annual Gross Output Value of Social Economy (10^8^ Yuan)	Its Growth Rate Under Every 5% Increase in Water Diversion Quantity from Transboundary River Basins
0%	21,454.98	–
5%	21,487.47	0.1514%
10%	21,519.89	0.1509%
15%	21,547.64	0.1290%
20%	21,563.77	0.0748%
25%	21,579.89	0.0748%
30%	21,595.98	0.0745%
35%	21,600.6	0.0214%
40%	21,600.6	0.0000%

## Abbreviations

FAO	Food and Agriculture Organization of the United Nations
GDP	Gross Domestic Product
RMB	Renminbi, Chinese yuan
CNY	Renminbi, Chinese yuan
PCR	the Rate of Population Change
PIGR	the Rate of Primary Industrial Production Growth
SIGR	the Rate of Secondary Industrial Production Growth
TIGR	the Rate of Tertiary Industrial Production Growth
